# Supplementary material for: Structural change as a key component for agricultural non-CO2 mitigation efforts
Source: Nat Commun. 2018 Mar 13;9:1060. doi: 10.1038/s41467-018-03489-1 (PMC5849693; doi:10.1038/s41467-018-03489-1)
Supplement: Supplementary file 1 — Supplementary Information [file 41467_2018_3489_MOESM1_ESM.pdf]

Frank et al. Supplementary Material - Structural change as a key component for agricultural non-CO_2 mitigation efforts

SUPPLEMENTARY FIGURES

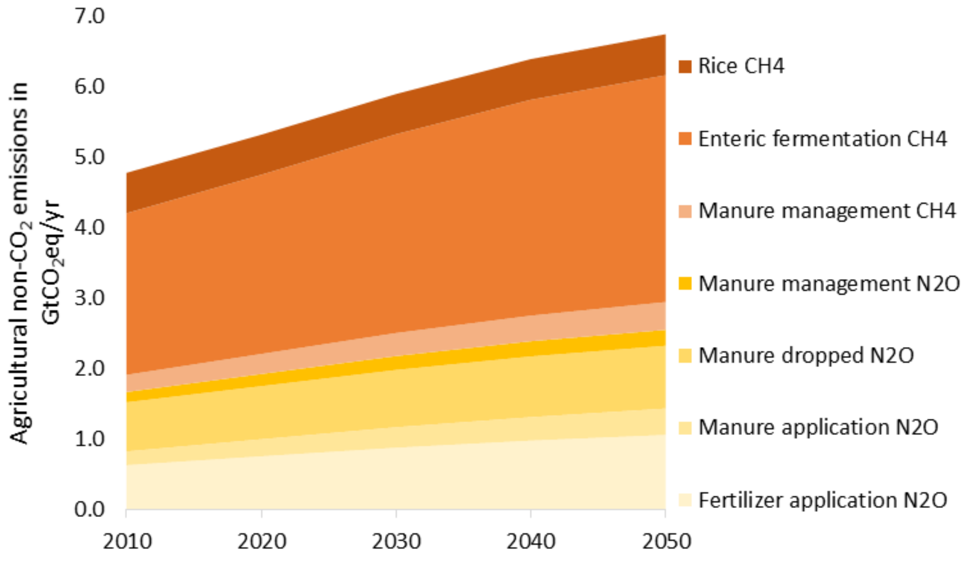

Supplementary Figure 1. Development of agricultural non-CO_2 emissions in the baseline scenario by GHG source.

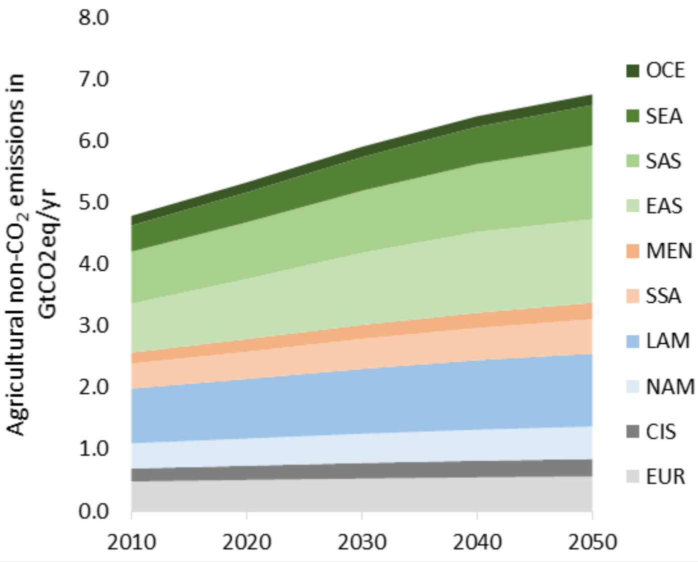

Supplementary Figure 2. Development of agricultural non-CO_2 emissions in the baseline scenario by region.

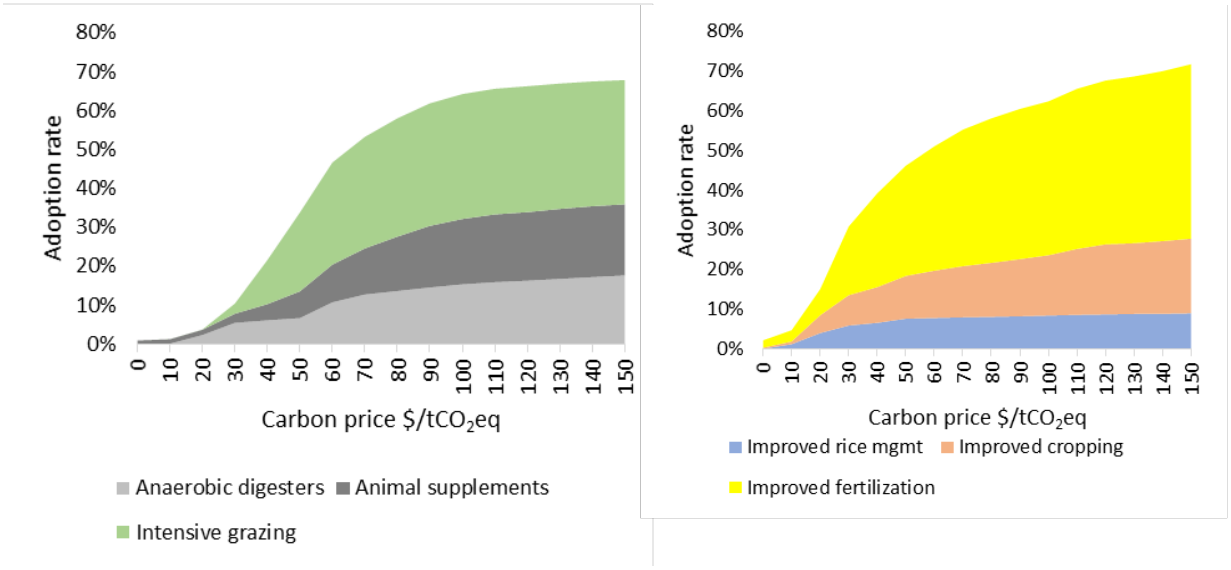

Supplementary Figure 3. Adoption rate of technical crop- and livestock mitigation options across carbon price scenarios in 2050.

SUPPLEMENTARY TABLES

Supplementary Table 1. Global average GHG reduction, impact on productivities and costs for technical mitigation options. Ranges across regions are presented in brackets.

Mitigation option	Non-CO_2 reduction	Productivity changes	Annual costs
Antibiotics[a]	*-2 (-6 to 0)%*	*+5%*	*6 (5 to 10) $/TLU*
Bovine somatotropin (bST)[b]	*+5 (0 to +10)%*	*+12 (11 – 13)%*	*110 (100 to 240) $/TLU*
Propionate precursors	*-13 (-10 to -19)%*	*+5%*	*41 (35 to 60) $/TLU*
Anti-methanogen vaccination	*-10 %*	*+5%*	*9 (5 to 20) $/TLU*
Intensive grazing	*-14 (-13 to -15)%*	*-11%*	*6 (5 to 20) $/TLU*
Large-scale complete-mix digesters	*-85%*	-	*25 (5 to 55) $/TLU*

Large-scale covered lagoon	*-85%*	*-*	*34 (10 to 70) $/TLU*
Large-scale fixed-film digester	*-85%*	*-*	*34 (10 to 60) $/TLU*
Large-scale plug-flow digesters	*-85%*	*-*	*38 (10 to 75) $/TLU*
Small-scale digester	*-50%*	*-*	*7 (5 to 15) $/TLU*
Centralized digester[c]	*-90%*	*-*	*8 (5 – 45) $/TLU*
No-till adoption	*-13 (-2 to -22)%*	*-6 (-22 to +1)%*	*-23 (-85 to 0) $/ha*
Optimal N fertilization	*+47 (-75 to +290)%*	*+167 (+1 to +775)%*	*16 (10 to 15) $/ha*
Split N fertilization	*-3 (-11 to +1)%*	*+3 (0 to +29)%*	*1 $/ha*
Nitrification inhibitors	*-7 (-2 to -23)%*	*+0.2 (-9 to +9))%*	*23 (15 to 35) $/ha*
100% residue incorporation	*+26 (+4 to +52)%*	*+5 (0 to +21)%*	*5 (0 to 30) $/ha*
Improved rice management: various combinations of different water-, residue-, and fertilizer management	*-100% to +300%*	*-33% to +47%*	*-16 to 123 $/ha*

TLU: livestock unit, an animal of 250 kg live weight. [a] Antibiotics: No application in Europe and Taiwan (Maron et al., 2013); [b] bST: No application in Australia, Canada, Europe, Japan, New Zealand (Dervilly-Pinel et al., 2014); [c] Centralized digesters are only applied in Europe;

SUPPLEMENTARY REFERENCES

Dervilly-Pinel, G., S. Prévost, F. Monteau and B. Le Bizec (2014). "Analytical strategies to detect use of recombinant bovine somatotropin in food-producing animals." TrAC Trends in Analytical Chemistry **53**(0): 1-10.

Maron, D., T. Smith and K. Nachman (2013). "Restrictions on antimicrobial use in food animal production: an international regulatory and economic survey." Globalization and Health **9**(1): 48.
